# Development and validation of experimental induction tasks for worry and rumination: A comparison of personalized and scripted approaches

**DOI:** 10.1016/j.janxdis.2026.103148

**Published:** 2026-03-12

**Authors:** Hanjoo Kim, Michelle G. Newman

**Affiliations:** a Department of Psychiatry, University of Michigan, MI, USA; b Department of Psychology, Pennsylvania State University, PA, USA

**Keywords:** Repetitive negative thinking (RNT), Worry, Rumination, Induction, Generalized anxiety disorder (GAD), Depression

## Abstract

**Background:**

Worry and rumination are two forms of repetitive negative thinking. Whereas prior research has highlighted both their distinct and overlapping characteristics, the experimental induction of these states provides a valuable means of investigating their mechanisms. Two induction methods have been used: personalized based on self-relevant content, and scripted using standardized prompts. However, no studies have directly compared these methods, and it remains unclear whether they elicit equally pronounced responses. Additionally, the moderating role of symptom profiles, such as elevated anxiety or depression symptoms, has not been well characterized.

**Method:**

This study systematically compared personalized and scripted induction methods for eliciting worry and rumination, and whether outcomes varied across induction focus (worry vs. rumination) and symptom-based groups. A total of 355 participants were categorized into three groups: individuals meeting the GAD-Q-IV criteria for generalized anxiety (*n* = 118), individuals with elevated depression symptoms on the BDI-II (*n* = 113), and individuals with low symptoms (*n* = 124). Participants were assigned to one of four conditions (personalized vs. scripted × worry vs. rumination).

**Results:**

Personalized induction methods elicited the targeted cognitive states more effectively than scripted methods, regardless of group. Additionally, the effect was strongest for target-specific outcomes relative to non-target outcomes. Results were robust to dimensional symptom modeling and demographic covariate adjustment.

**Conclusions:**

These findings highlight that personalized induction methods may provide a more ecologically valid and responsive tool than scripted induction approaches for experimentally eliciting worry and rumination. Implications for induction selection and study design are discussed.

## 1. Introduction

Worry and rumination are two forms of repetitive negative thinking that have been experimentally shown to cause emotional distress at both the subjective (e.g., Jamil & Llera, 2021; Kim & Newman, 2022; Llera & Newman, 2014; Stapinski et al., 2010) and physiological levels (e.g., Hofmann et al., 2005; Llera & Newman, 2010; Ottaviani et al., 2016; Steinfurth et al., 2017). Worry involves excessive and uncontrollable thoughts about potential future threats (Borkovec et al., 1983), whereas rumination entails persistent focus on the causes and consequences of problems and associated emotions (Nolen-Hoeksema, 1991). Worry is considered a defining feature of generalized anxiety disorder (GAD; Newman et al., 2013), whereas rumination is closely associated with major depressive disorder (MDD; Nolen-Hoeksema et al., 2008).

Unlike worry, the conceptualization of rumination has evolved over time, with increasing recognition that it comprises multiple subcomponents rather than a single, homogeneous process. Early work primarily emphasized depressive rumination/brooding as a characteristic response style in the context of depressed mood, involving reflective self-focus on symptoms, causes, and consequences of distress (Nolen-Hoeksema, 1991; Treynor et al., 2003). In contrast, more recent frameworks have more explicitly distinguished rumination from worry by defining it as past-focused repetitive negative thinking that can occur across emotional states and diagnostic categories (Ehring & Watkins, 2008; Kingston et al., 2014; Nolen-Hoeksema et al., 2008; Watkins, 2008). This distinction has important implications for experimental induction paradigms, as procedures originally developed to capture depressive response styles in depressed populations may not reliably elicit highly salient or negatively valenced rumination in non-dysphoric or mixed-symptom samples.

Despite these conceptual distinctions, worry and rumination show substantial overlap across studies (Erickson et al., 2020; Watkins et al., 2005). Measures of worry and rumination are often highly correlated in both clinical and non-clinical populations (Segerstrom et al., 2000; Stade & Ruscio, 2022; Szkodny & Newman, 2019). Some studies suggest that worry and rumination may share common latent structures (Segerstrom et al., 2000; Topper et al., 2014). Moreover, both forms of repetitive thinking are prevalent across other mental health conditions, such as posttraumatic stress disorder, social anxiety disorder, and bipolar disorder (Hearn et al., 2017; Mellings & Alden, 2000; Moulds et al., 2020; Sarisoy et al., 2014; Silveira & Kauer-Sant’Anna, 2015; Tull et al., 2011), highlighting their potential roles as transdiagnostic mechanisms of emotion dysregulation.

Research has also documented meaningful points of divergence. Worry has tended to involve cognitive biases away from threat, whereas rumination was biased toward themes of loss and failure (Hur et al., 2019). Physiologically, worry was associated with lower heart rate variability, whereas rumination showed a comparatively weaker link to this marker (Aldao et al., 2013). In terms of cognitive appraisals, worry was linked to low perceived coping efficacy and problem-solving impairment (Llera & Newman, 2020), whereas rumination was often tied to problem-solving avoidance (Hong, 2007). These differences underscore the importance of further research aimed at disentangling their shared and unique influences.

Understanding these intricacies is essential for developing targeted interventions. Laboratory-based studies offer a controlled environment to experimentally manipulate and compare the effects of worry and rumination. A common approach was to use induction paradigms that elicited these mental states, but a key methodological challenge has been the lack of consistency across induction methods, complicating direct comparisons.

Worry inductions typically used personalized induction prompts, where participants were asked to reflect on personally relevant worries, an approach originally developed by Borkovec and Inz (1990) and widely adopted in experimental research (e.g., Behar et al., 2005; Fisher & Newman, 2013; Karim et al., 2017; Llera & Newman, 2010, 2014, 2020; Oathes et al., 2008). In contrast, rumination is most often induced using scripted procedures, in which participants read standardized prompts designed to evoke ruminative thoughts, following the method introduced by Nolen-Hoeksema and Morrow (1993), and subsequently used in many studies (e.g., LeMoult & Joormann, 2014; Watkins & Brown, 2002; Whitmer & Gotlib, 2012). Although the scripted rumination induction has proven useful for examining reflective cognitive styles, it remains unclear whether it effectively elicits the more emotionally intense aspects of depressive rumination, particularly in short-term laboratory paradigms.

Experimental comparisons of worry and rumination have generally adopted one of two approaches: either using personalized inductions for both processes (e.g., Jamil & Llera, 2021; Kim & Newman, 2022, 2023; McLaughlin et al., 2007), or relying on scripted inductions (e.g., Capobianco et al., 2018; Lewis et al., 2019). However, to the best of our knowledge, no studies have systematically examined the rationale for selecting one method over the other, and no clear consensus has emerged regarding which method is more suitable. Furthermore, it remains unclear whether differences in responses to these inductions are moderated by participants’ baseline characteristics, such as anxiety or depression levels.

To address these gaps, the present study compared personalized and scripted induction procedures for worry and rumination, while also examining whether induction effects generalized across clinically relevant diagnostic groups. Although the primary focus of the study was on transdiagnostic cognitive processes rather than diagnostic group differences per se, diagnostic grouping was used to ensure representation of individuals with elevated worry- and rumination-related symptom profiles and to evaluate whether the effectiveness of induction procedures varied as a function of clinical status. Specifically, we sought to answer the following three research questions (RQs):

RQ 1. Which induction method, personalized or scripted, elicits a stronger targeted response under controlled conditions? RQ 2. Do worry and rumination inductions show greater effects on their targeted cognitive processes than on non-target processes (convergent vs. discriminant effects)? RQ 3. To what extent do diagnostic groups (GAD, Depression, and low anxiety/depression symptom group) moderate the impact of each induction method?

To further evaluate the robustness of the primary findings, we examined three complementary sensitivity questions: Sensitivity Analysis 1. whether the observed induction effects remained robust after adjustment for demographic covariates; Sensitivity Analysis 2. whether rumination-related effects were different within the depression subgroup, consistent with the original conceptualization of scripted rumination paradigms as modeling depressive response styles; Sensitivity Analysis 3. whether similar patterns were observed when symptom severity was modeled dimensionally rather than categorically.

## 2. Method

### 2.1. Participants

A total of 356 individuals were recruited from a university subject pool. Due to a computer error, data from one participant was excluded, resulting in a final sample of 355 participants. These included 118 individuals with elevated generalized anxiety disorder symptoms and low depressive symptoms (GAD group), 113 with elevated depressive symptoms and low generalized anxiety symptoms (Depression group), and 124 low anxiety/depression symptom group (LS group). Participants were assigned to the GAD group if they met full diagnostic criteria on the Generalized Anxiety Disorder Questionnaire-IV (GAD-Q-IV; Newman et al., 2002) and scored 13 or below on the Beck Depression Inventory-II (BDI-II; Beck, Steer, & Brown, 1996). The depression group included participants who scored 20 or above on the BDI-II and did not meet diagnostic criteria on the GAD-Q-IV. The LS group consisted of individuals who neither met the GAD criteria nor scored 14 or higher on the BDI-II. Participants were assigned to one of four experimental conditions (personalized worry, personalized rumination, scripted worry, scripted rumination) using stratified randomization by diagnostic group (GAD, Depression, LS).^3^ A pre-generated counterbalancing list with randomized block order was used to ensure equal allocation across conditions within each group. Assignment was handled by a separate scheduling staff member who was not involved in data collection, and experimenters conducting the sessions were blind to participants’ diagnostic group status. Participant demographics are presented in Table 1. The sample was predominantly female (282 women, 79.4%), with a mean age of 18.23 years (*SD* = 2.83). The majority identified as White (75.2%), followed by Asian (10.4%), Hispanic (6.2%), Black or African American (5.1%), and other racial backgrounds (3.1%). Participants exhibited varying levels of anxiety and depressive symptoms (GAD-Q-IV: *M* = 4.88, *SD* = 3.88; BDI-II: *M* = 12.35, *SD* = 10.42). A more detailed description of the recruitment and screening procedures can be found in Method S1.

### 2.2. Procedure

This study was approved by the Institutional Review Board of the authors’ affiliated institution. Prior to participation, all individuals provided written informed consent. At the outset of the experiment, the concepts of worry and rumination were introduced. Consistent with definitions by Borkovec et al. (1983) and Nolen-Hoeksema (1991), and incorporating temporal distinctions emphasized in later theoretical frameworks (e.g., Ehring & Watkins, 2008; Nolen-Hoeksema et al., 2008; Watkins, 2008), worry was described as “a chain of uncontrollable thoughts and images about things that might happen in the future,” accompanied by illustrative examples emphasizing future-oriented concerns. In contrast, rumination was defined as “passively and repetitively thinking about possible causes, implications, and consequences of stressful events and negative feelings, as opposed to their solutions,” with examples focusing on past negative experiences (Method S2). All experimental sessions were conducted in the same laboratory testing room. Participants were tested individually in one-on-one sessions, with only one participant present at a time. The testing environment was standardized across sessions, including consistent room temperature and lighting conditions, and the laboratory room contained no windows, minimizing external visual distractions and environmental variability. The experiment began with a 5-minute resting baseline period to allow participants to acclimate to the environment. Participants were then assigned to one of four thought induction conditions. In the personalized induction conditions, participants first generated five personally relevant worry- or rumination-related scenarios. These were evaluated and pre-screened for eligibility prior to the experimental induction. Participants then vividly focused on their most worrisome or ruminative personal topic for 2 min. In the scripted induction conditions, participants read a series of prewritten worry- or rumination-related statements at their own pace for 8 min. Following both the baseline and induction phases, participants rated their current levels of worry and rumination. Other than the induction-method features themselves, procedures were held constant across conditions. Specifically, participants received the same definitions of worry and rumination, were tested individually in the same laboratory environment, completed the same baseline period, and provided ratings of current worry and rumination at the same assessment points. Thus, the conditions differed only in features inherent to the induction methods being compared, including induction format (personalized self-generated topic vs. scripted statements), the presence of a preparatory topic generation/screening phase for the personalized induction, and induction duration (2 min vs. 8 min). After the experiment, participants received research credit for their involvement. All procedures were administered using E-Prime 2.0 software (Psychology Software Tools inc, 2002). To facilitate replication and adaptation of the induction procedures in future research, we provide detailed experimenter instructions, participant prompts, scenario screening criteria, and scripted induction texts in the Supplementary Materials (Method S3).

### 2.3. Instruments

#### 2.3.1. Generalized anxiety disorder questionnaire-IV (Newman et al., 2002)

The GAD-Q-IV is a nine-item self-report measure that assesses symptoms of GAD based on the diagnostic criteria outlined in the Diagnostic and Statistical Manual of Mental Disorders (American Psychiatric Association, 2013). Diagnoses can be determined either dimensionally or by evaluating whether participants meet full diagnostic criteria. For the primary analyses, we employed criterion-based scoring, as recommended by Moore et al. (2014), which demonstrated higher sensitivity (89%) and specificity (82%) compared to dimensional scoring. Internal consistency of the current sample was strong (Cronbach’s α =.82).^4^

#### 2.3.2. Beck depression Inventory-II (Beck, Steer, & Brown, 1996)

The BDI-II is a 21-item self-report questionnaire designed to assess the severity of depressive symptoms. It has shown strong convergent and discriminant validity (Beck, Steer, & Brown, 1996; Steer et al., 1999) and high retest reliability (Beck et al., 1988). A cutoff score of 18 has been associated with high sensitivity (94%) and specificity (92%) for detecting depression (Arnau et al., 2001). For this study, we applied a stricter cutoff to classify participants: scores ≥ 20 for moderate to severe depression and ≤ 13 for minimal symptoms, in accordance with prior recommendations (Beck, Steer & Ball, & Ranieri, 1996). Internal consistency in our sample was excellent (Cronbach’s α =.92).

#### 2.3.3. Worry and rumination likert-type scales

Participants rated their levels of worry and rumination on a 9-point Likert-type scale ranging from 0 (“not at all”) to 8 (“extremely”) at two time points: following the resting baseline and after the induction phase.

#### 2.3.4. Personalized induction methods

The personalized worry and rumination inductions were adapted from the method used by Llera & Newman, (2010), (2014). As with prior studies using this method (e.g., Jamil & Llera, 2021; Kim & Newman, 2022, 2023), this entailed a pre-selection of personally relevant topics and a practice phase to ensure relevance and saliency. Thus, prior to the induction phase, participants were asked to generate five personally relevant worry- or rumination-related scenarios. They then practiced thinking about each scenario for one minute and rated its intensity and temporal orientation on the 9-point Likert-type scale described above. These ratings were then used for standardized pre-screening. A scenario qualified for the induction phase if the self-reported intensity for the target thought process (worry or rumination) was greater than 4 and at least 3 points higher than the non-target process. In addition, the temporal orientation of the scenario had to match the induction focus: future-oriented (score > 4) for worry and past-oriented (score < 4) for rumination. All participants were able to generate at least one scenario that met these criteria, and no participants were excluded due to failure to produce qualifying scenarios. During the induction phase, participants focused on their most intense validated scenario for 2 min. This brief imagery-based engagement is consistent with prior personalized induction paradigms emphasizing focused engagement with emotionally salient content rather than prolonged exposure to standardized prompts (e.g., Llera & Newman, 2010, 2014; McLaughlin et al., 2007). Further methodological details are provided in Method S3.

#### 2.3.5. Scripted Induction Methods

For the scripted worry induction, participants read items drawn from the Worry Domains Questionnaire (WDQ; Tallis et al., 1994), a 25-item measure adapted from the General Worry Questionnaire (GWQ; Tallis et al., 1992). The WDQ includes a broad range of common worry topics, such as interpersonal problems, future uncertainty, work-related concerns, and financial issues. Consistent with prior studies using this approach (e.g., Freeman et al., 2013; Ikani et al., 2022), participants were instructed to read through these items at their own pace. The scripted rumination induction was based on the procedure developed by Nolen-Hoeksema and Morrow (1993), which includes 45 statements designed to elicit self-reflective thinking about current emotional states and personal characteristics. In the original Nolen-Hoeksema and Morrow (1993)’s paradigm, participants were instructed to engage in rumination for eight minutes, a duration shown to reliably induce mood changes among depressed individuals. The present study preserved this duration for the scripted rumination condition. To facilitate comparability across induction conditions, the scripted worry induction also used an 8-minute duration. Additional procedural details are available in Method S4.

### 2.4. Analytic plan

All analyses were conducted using R version 4.5.1 (R Core Team, 2025). Descriptive statistics were computed for demographic characteristics, as well as GAD-Q-IV and BDI-II scores. Group and condition differences were examined using ANOVAs and chi-square tests, with pairwise comparisons conducted as appropriate. To evaluate the effects of induction method (personalized vs. scripted) and induction focus (worry vs. rumination), we fit random-intercept linear mixed models with time (baseline vs. induction phase) as a within-subject factor (RQ1 and RQ2). To address RQ3, we conducted an extended analysis by including group (GAD, Depression, LS) as an additional between subject factor. We focused on the interactions between induction method or focus and time, as well as their three-way interactions with group. For significant three-way interactions, simple slopes were estimated using Howell (2012)’s method to assess within-condition changes and between-condition differences in slopes.

To evaluate the robustness of the primary findings, we conducted three sensitivity analyses. First, we re-estimated the primary models with additional adjustment for demographic covariates, including age, gender, and race (Sensitivity Analysis 1). Second, to further characterize disorder-specific effects and to increase sensitivity to rumination-related responses that may not have been detectable in higher-order interaction models, we performed targeted follow-up analyses restricted to the depression group, directly examining the effects of the rumination induction within this subsample (Sensitivity Analysis 2). Finally, as an additional robustness check, we re-estimated the models by replacing the categorical diagnostic group variable with continuous symptom severity measures (GAD-Q-IV and BDI-II scores), which may have offered greater statistical sensitivity than categorical classifications (Ruscio, 2019; Sensitivity Analysis 3).

## 3. Results

### 3.1. Descriptive statistics

As part of the baseline checks, continuous symptom severity measures (GAD-Q-IV and BDI-II) were used to assess both randomization balance across conditions and the validity of diagnostic group characterization. Consistent with the group assignment criteria, the GAD group scored higher on the GAD-Q-IV than both the depression and LS groups, whereas the depression group scored higher than the LS group (all *ps* <.001). On the BDI-II, the depression group scored higher than both the GAD and LS groups, and the GAD group scored higher than the LS group (all *p* < .001). There were no significant group differences in age, gender distribution, or racial identity (Table 1).

In addition, no significant differences were observed across experimental conditions in age, *F*(3, 170.75) = 2.22, *p* = .087, partial *η*^*2*^ = .04; gender, χ^2^(3) = 1.16, *p* = .762, *V* = .06; or racial identity, χ^2^(12) = 15.42, *p* = .219, *V* = .12. Similarly, GAD-Q-IV scores, *F*(3, 194.85) = 1.61, *p* = .189, partial *η*^*2*^ = .02; and BDI-II scores, *F*(3, 194.36) = .37, *p* = .774, partial *η*^*2*^ = .01 did not significantly differ across conditions. Thus, participants were well-balanced across experimental conditions.

### 3.2. Main outcomes

**Fig. 1.**
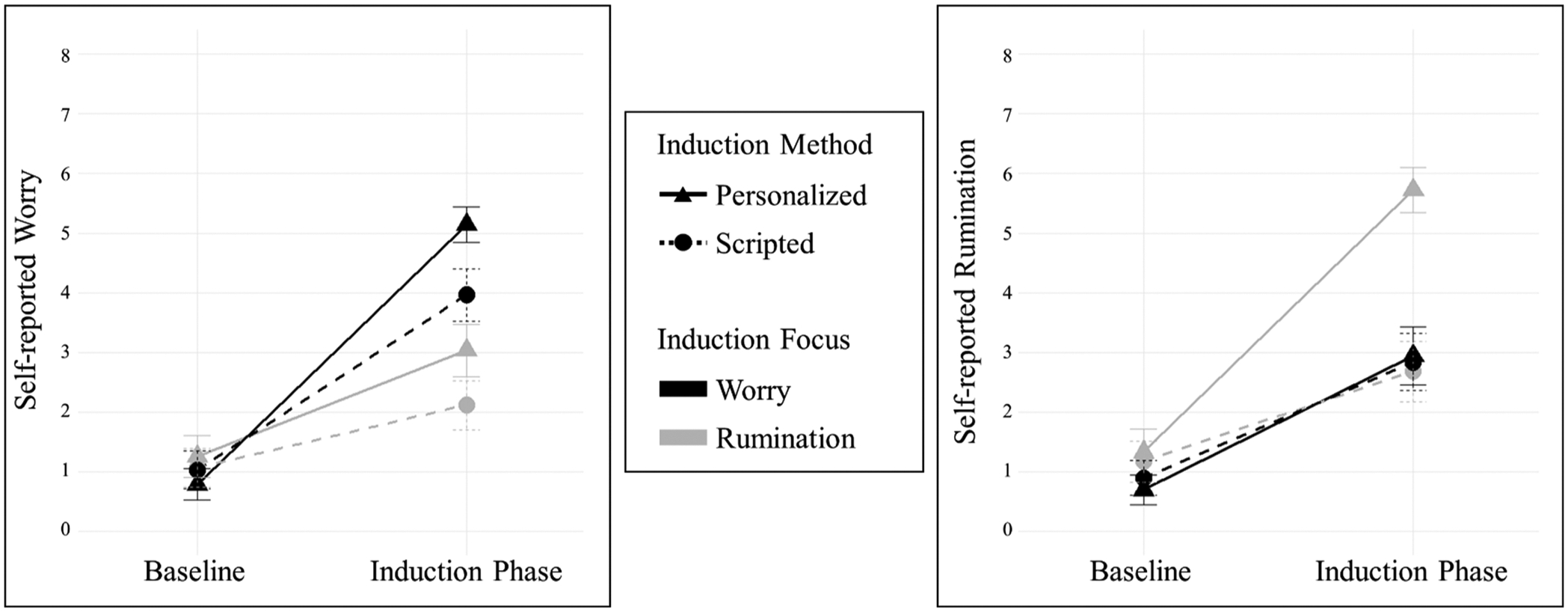
Changes in self-reported worry (left) and rumination (right) by induction method (personalized vs. scripted) and induction focus (worry vs. rumination). *Note*. Error bars indicate 95% confidence intervals.

Table 2 presents the means and standard deviations of self-reported worry and rumination across all conditions. Interaction patterns are visualized in Fig. 1.

#### 3.2.1. RQ1: Effects of induction method (personalized vs. scripted)

For both worry, *F*(1, 181.68) = 22.41, *p* < .001, partial *η*^*2*^ = .11; and rumination inductions, *F*(1, 176.87) = 73.30, *p* < .001, partial *η*^*2*^ = .29, there were significant two-way interactions between induction method and time. Both the personalized, *B* = 4.36, *SE* = .18, *p* < .001; and scripted worry inductions, *B* = 2.93, *SE* = .24, *p* < .001 significantly increased self-reported worry from the resting baseline. Similarly, both the personalized, *B* = 4.39, *SE* = .27, *p* < .001; and scripted rumination inductions, *B* = 1.51, *SE* = .20, *p* < .001 increased rumination. However, the personalized worry induction led to greater increased worry, *t* (88) = 4.81, *p* < .001, *d* = .36; and the personalized rumination induction led to greater increased rumination, *t*(85) = 8.60, *p* < .001, *d* = .65 than their scripted counterparts (Fig. 1 and Table S2).

#### 3.2.2. RQ2: Effects of induction focus (worry vs. rumination)

For the personalized inductions, there was an interaction between induction focus (worry vs. rumination) and time, indicating differential effects on self-reported worry, *F*(1, 178) = 66.02, *p* < .001, partial *η*^*2*^ = .27; and rumination, *F*(1, 178) = 36.62, *p* < .001, patial *η*^*2*^ = .17. With respect to convergent validity, both personalized worry (*B* = 4.36, *SE* =.18, *p* < .001) and personalized rumination (*B* = 4.39, *SE* =.27, *p* < .001) inductions increased their respective target processes from baseline. With respect to discriminant validity, the personalized worry induction increased worry more from baseline than the personalized rumination induction (*B* = 1.78, *SE* =.26, *p* < .001), *t*(89) = 8.13, *p* < .001, *d* = .61. Also, the personalized rumination induction increased rumination more from baseline than the personalized worry induction (*B* = 2.24, *SE* =.24, *p* < .001), *t*(89) = 6.05, *p* < .001, *d* = .45 (Fig. 1 and Table S3).

For scripted inductions, there was a significant two-way interaction between induction focus (scripted worry vs. scripted rumination) and time on self-reported worry, *F*(1, 173) = 39.73, *p* < .001, partial *η*^*2*^ = .19; but not on self-reported rumination, *F*(1, 173) = 2.07, *p* = .153, partial *η*^*2*^ = .01. Regarding convergent validity, the scripted worry induction (*B* = 2.93, *SE* =.24, *p* < .001) and the scripted rumination induction (*B* = 1.51, *SE* =.20, *p* < .001) were each associated with significant increases in their respective target processes. Regarding discriminant validity, the scripted worry induction produced a significantly greater increase in worry from baseline than the scripted rumination induction (*B* = 1.05, *SE* =.18, *p* < .001), *t*(85) = 6.33, *p* < .001, *d* = .48. In contrast, no significant difference was observed between the scripted rumination and the scripted worry inductions (*B* = 1.94, *SE* =.22, *p* < .001) for rumination, *t*(85) = −1.44, *p* = .154, *d* = .11 (Fig. 1; Table S4).

#### 3.2.3. RQ3: Group differences in induction effects

There were no significant induction method × time × group interactions for self-reported worry, *F*(2, 178.86) = 2.49, *p* = .086, partial *η*^2^ = .03; or rumination, *F*(2, 174.12) = 2.37, *p* = .096, partial *η*^2^ = .03. Likewise, there were no induction focus × time × group interactions within either the personalized inductions (worry: *F*(2, 174) = .53, *p* = .587, partial *η*^2^ = .01; rumination: *F*(2, 174) = 1.84, *p* = .161, partial *η*^2^ = .02) or the scripted inductions (worry: *F*(2, 169) = 2.78, *p* = .065, partial *η*^2^ = .03; rumination: *F*(2, 169) = 1.29, *p* = .277, partial *η*^2^ = .02). Across all models, the two-way interactions between induction method and time, as well as between induction focus and time, remained statistically significant and directionally consistent with analyses described above following the inclusion of group as a moderator. The significance and directionality of these effects can be directly verified from the fixed-effect estimates presented in Tables S5–S7.

#### 3.2.4. Sensitivity Analysis 1: Adjustment for demographic covariates

Inclusion of demographic covariates did not alter the statistical significance or direction of any primary induction effects or higher-order interaction terms. Across all models, the core findings corresponding to RQ1-RQ3 remained fully consistent with the primary analyses. Detailed fixed-effect estimates documenting these retained patterns are presented in Tables S8–S13.

#### 3.2.5. Sensitivity Analysis 2: Depression group restricted subsample analysis

Within the depression subgroup, there was a significant interaction between induction method and time, indicating differential pre-to-post changes across induction methods, *F*(1, 55.84) = 19.25, *p* < .001, partial *η*^2^ = .26. Both the scripted (*B* = 1.71, SE = 0.36, *p* < .001) and personalized rumination inductions (*B* = 4.29, SE = 0.47, *p* < .001) increased rumination from baseline. However, the personalized induction increased rumination more than the scripted induction, *t*(27) = 4.37, *p* < .001, *d* = 0.60. Thus, the superiority of the personalized induction over the scripted method was preserved within the depression group. The corresponding fixed-effect estimates are presented in Table S14.

For depressed participants in the scripted conditions, there was no significant interaction between induction focus and time, *F*(1, 56) = 2.80, *p* = .100, *η*^2^ = .05. Both the scripted worry (*B* = 2.67, SE = 0.44, *p* < .001) and scripted rumination inductions (*B* = 1.71, SE = 0.36, *p* < .001) increased self-reported rumination. Consistent with full-sample findings, however, the scripted rumination induction did not increase self-reported rumination more than the scripted worry induction, indicating comparatively weaker discriminant validity, *t*(27) = 1.69, *p* = .103, *d* = .23. Fixed-effect estimates for this model are reported in Table S15.

#### 3.2.6. Sensitivity Analysis 3: Continuous symptom severity models

Most three-way interactions involving symptom severity (GAD-Q-IV or BDI-II), experimental condition (induction method or induction focus), and time were nonsignificant, consistent with the original group-based models. Only two significant three-way interactions emerged with small moderation effects, and the personalized induction generally remained more effective than scripted induction. First, anxiety severity significantly moderated rumination induction effects on reported rumination across induction methods (GAD-Q-IV × induction method × time), *F*(1, 175.45) = 6.22, *p* = .014, partial *η*^2^ = .03 (see Table S16 for the corresponding fixed-effect estimates). At low (−1 *SD*; personalized: *B* = 4.75, *SE* =.37, *p* < .001; scripted: *B* = 1.04, *SE* =.28, *p* < .001) and high (+1 *SD*; personalized: *B* = 4.02, *SE* =.38, *p* < .001; scripted: *B* = 1.97, *SE* =.28, *p* < .001) anxiety severity, both induction methods significantly increased rumination. Nonetheless, the personalized induction yielded larger pre-to-post increased rumination than the scripted induction at both low, *t*(84) = 7.95, *p* < .001, *d* = .61, and high anxiety severity, *t*(84) = 4.38, *p* < .001, *d* = .33. Thus, the overall superiority of the personalized method for rumination induction was preserved across anxiety severity levels.

Second, depressive symptom severity significantly moderated worry induction effects on reported worry across induction methods (BDI-II × induction method × time), *F*(1, 180.45) = 4.74, *p* = .031, partial *η*^2^ = .03 (see Table S19 for the corresponding fixed-effect estimates). At low (−1 *SD*; personalized: *B* = 4.59, *SE* = 0.27, *p* < .001; scripted: *B* = 2.50, *SE* = 0.32, *p* < .001) and high (+1 *SD*; personalized: *B* = 4.12, *SE* = 0.27, *p* < .001; scripted: *B* = 3.35, *SE* = 0.31, *p* < .001) depressive symptom severity, both induction methods significantly increased worry. At the same time, at low depressive severity, the personalized (vs. scripted) induction produced a significantly larger pre-to-post increase in worry, *t*(87) = 5.05, *p* < .001, *d* = .38. However, at high depressive symptom severity, the difference between worry induction methods was no longer significant, *t*(87) = 1.86, *p* = .067, *d* = .14. The reduced between-method difference at higher depressive symptom severity was driven primarily by a relatively larger increase in the scripted worry induction slope, rather than by a reduction in the personalized induction effect.

## 4. Discussion

This study compared the effectiveness of personalized and scripted methods for inducing worry and rumination, an area that has received limited empirical attention. We also examined whether these methods produced target-specific versus cross-process effects on self-reported worry and rumination, and whether these effects differed across diagnostic groups. Our findings demonstrated that the personalized induction method consistently outperformed the scripted method in eliciting the intended repetitive thought process, regardless of group. In addition, the personalized worry induction increased worry more than the personalized rumination induction, and the personalized rumination induction increased rumination more than the personalized worry induction. Together, this provides support for the construct validity of the personalized induction procedures.

In contrast, the scripted methods showed weaker target-specific differentiation, particularly for rumination. Although the scripted worry induction increased worry more than the scripted rumination induction, the scripted rumination induction did not have a stronger effect on increased rumination than the scripted worry induction. This pattern may stem from differing intent behind the development of the scripted rumination induction. Whereas the personalized worry induction by Borkovec and Inz (1990) was specifically designed to reliably elicit anticipatory worry in both anxious and non-anxious individuals, the scripted rumination induction developed by Nolen-Hoeksema and Morrow (1993) was intended to examine the effects of reflective thinking on depressed mood in individuals with depression. As such, the script was deliberately emotionally neutral, focusing on self-reflection rather than overtly negative content.

This methodological feature may explain its applicability to studies aiming to investigate reflective forms of rumination. However, in the context of experimental designs attempting to manipulate more negatively valenced repetitive thinking, this approach may be less likely to elicit self-critical and negative emotional features commonly associated with depressive rumination/brooding (Conway et al., 2000; Papageorgiou & Wells, 2004; Robinson & Alloy, 2003). Furthermore, the emotionally neutral nature of the scripted rumination induction may have contributed to its limited effectiveness, as it did not elicit greater self-reported rumination than the scripted worry condition.

A series of sensitivity analyses were conducted to evaluate the robustness of the primary findings. For example, including age, gender, and race as covariates (Sensitivity Analysis 1) did not alter the primary findings, indicating that induction effects were not attributable to demographic confounding within the demographic limits of this sample. Also, we conducted analyses focused solely on the depression group for whom the scripted rumination induction was developed (Sensitivity Analysis 2). However, isolating this subgroup did not reveal stronger or qualitatively different effects for the scripted rumination induction. The personalized induction continued to elicit substantially larger changes than the scripted induction and better discriminant validity. This suggests that the lower performance of the scripted rumination paradigm may be attributable to features of the induction procedure itself rather than to a mismatch between induction content and participants’ diagnostic status.

The dimensional sensitivity analyses (Sensitivity Analysis 3), which replaced diagnostic group with continuous symptom severity measures, largely converged with the original group-based models across both induction method and induction focus comparisons. Most three-way interactions were not significant. In addition, even though two three-way interactions were significant on cross domain outcomes (anxiety severity moderating rumination induction and depression severity moderating worry induction), these analyses mostly continued to find that the personalized (vs. scripted) inductions led to stronger effects. Only at high depression severity was there no significant difference between personalized and scripted worry inductions and this effect emerged only on worry. At the same time, this one effect was driven by variability in the scripted induction, whereas responses to the personalized induction remained uniformly strong and exhibited comparatively smaller proportional variation across severity levels.

Overall responsiveness in the scripted condition was lower than in the personalized condition, which may have rendered it more sensitive to individual differences that facilitated cross-domain emotional activation. In contrast, personalized induction methods appeared to elicit near-ceiling levels of engagement across participants, such that additional modulation by cross-domain symptom severity became less pronounced. These findings indicated that personalized induction methods exhibited greater stability across both categorical and dimensional analytic frameworks.

Taken together, the findings underscore the importance of how repetitive negative thinking is experimentally induced, as the choice of induction method can substantially influence observed outcomes. Given that worry and rumination are central transdiagnostic processes implicated in emotional disorders (Erickson et al., 2020; Ruscio et al., 2011), careful selection of induction procedures is essential for advancing causal research in this domain. Our results suggest that personalized induction methods, which were tailored to participants’ real-life concerns, may offer a particularly robust and ecologically valid approach for future experimental paradigms.

From a translational perspective, these findings have implications for both experimental paradigms and intervention-oriented research that rely on reliable elicitation of repetitive negative thinking. Personalized induction procedures may improve the precision with which maladaptive cognitive states are experimentally evoked, thereby enhancing the sensitivity of laboratory tasks used to probe neural, physiological, and behavioral mechanisms associated with worry and rumination. More robust and target-specific induction may also facilitate the development and evaluation of intervention components that aim to modify repetitive negative thinking, including task-based cognitive training, emotion regulation paradigms, and neuromodulation protocols that depend on state-dependent engagement of relevant neural circuits. By establishing the relative effectiveness and construct validity of different induction formats, the present findings provide a methodological foundation that can strengthen downstream translational applications without pre-supposing specific therapeutic mechanisms.

Several limitations should be noted. First, although GAD and depression were examined as distinct diagnostic groups in the present study, these disorders frequently co-occur. Future research including individuals with comorbid GAD and depression, and use of dimensional symptom profiles for both disorders, may help clarify whether our results are replicated within these samples. Second, we used self-report measures for diagnosis and findings may not generalize to individuals diagnosed with a structured interview. Third, the present study relied on a university student sample that was relatively homogeneous in age. Developmental differences in emotion regulation capacity, cognitive control, and habitual engagement in repetitive negative thinking may have influenced responsiveness to experimental induction procedures (McRae et al., 2012; Schweizer et al., 2020; Theurel & Gentaz, 2018). Given that the present sample primarily consisted of late adolescents and emerging adults, future research should examine the generalizability of these findings across broader age ranges. In addition, replication in samples with more diverse sociodemographic characteristics is needed to further evaluate robustness, and external validity.

Fourth, the present study relied on self-reported state worry and rumination ratings as the primary outcomes, which may have been influenced by expectancy effects and demand characteristics in experimental induction paradigms. However, the observed pattern of findings was unlikely to be fully attributable to expectancy-driven responding. Participants rated both worry and rumination at each assessment point, allowing for differential change patterns across constructs rather than relying on single-process endorsement. Moreover, consistent advantages of the personalized induction were observed across both worry and rumination outcomes and across distinct task formats, which would be less expected if responses primarily reflected compliance with perceived experimental demands. Nevertheless, future studies could further strengthen causal inference by incorporating complementary objective or implicit measures (e.g., physiological, behavioral, or neural indices) and additional blinding procedures when feasible.

Fifth, because we reproduced the separate induction methods consistent with prior experimental studies, and studies rarely examine both thought processes simultaneously, it is important to highlight how the inductions differed beyond the fact that the scripted induction required participants to read phrases intended to induce rumination/worry and the personalized induction had participants choose personally relevant topics. There were two additional differences between the two induction methods. One of these was that similar to other studies using the scripted method (e.g., Freeman et al., 2013; Ikani et al., 2022; Whitmer & Gotlib, 2012), there was no pre-selection and/or practice phase. However, the personalized induction method entailed a pre-selection of personalized topics (for worry/rumination) and a practice phase of those topics similar to other personalized induction studies (e.g., Kim & Newman, 2022, 2023; Llera & Newman, 2010, 2014). Although this might have contributed to its enhanced effects, we believe this is an important part of the personalized induction procedure and therefore would be part of what we would recommend researchers replicate. Nonetheless, a future study comparing the two methods could implement a practice phase in both inductions to reduce this potential limitation. There were also differences in duration of the induction period such that the personalized induction lasted 2 min and the scripted induction lasted 8 min. Accordingly, the present design assumed that these durations were optimized for each induction format. However, such differences may have introduced variability in engagement intensity and temporal dynamics that could have contributed to the observed effects alongside induction format. At the same time, introducing an arbitrary standardized duration across both inductions could be critiqued for not following the typical duration for that induction. Future studies could extend the present design by systematically manipulating induction duration and engagement demands to disentangle these factors and clarify their independent and interactive contributions. Finally, although the present study focused on worry and depressive rumination, future research should extend this methodological framework to other forms of repetitive negative thinking, including processes such as angry rumination and obsessive thought patterns (Gerin et al., 2006; Szkodny & Newman, 2019).

Although the use of a college student sample may raise questions about clinical relevance, epidemiological data support the importance of clinically screened college populations. Studies show that the 12-month prevalence of anxiety (15.7%−11.94%) and depression (5.9%−7.4%) disorders among college students is quite high (Auerbach et al., 2016; Blanco et al., 2008) and growing even stronger (Center for Collegiate Mental Health, 2023; Xiao et al., 2017), and that there is no difference in this prevalence between college students and same age individuals not in college (Blanco et al., 2008). Therefore, we argue that clinically screened college students are highly relevant.

As with college student samples, some people suggest that clinical relevancy requires that a sample be treatment seeking. We dispute this notion for several reasons. First, treatment seeking is not used as a criterion for estimating the prevalence of mental health disorders in any epidemiological study, including those cited above. These same studies find that most college students with mental disorders do not receive treatment (Auerbach et al., 2016; Olfson et al., 2019) and these disparities are even higher among college student minorities (Hunt et al., 2015; Lipson et al., 2018). Thus, we argue that treatment seeking should not be a criterion for clinical relevancy in psychology studies.

Another potential limitation was that information regarding current psychotropic medication use and psychotherapy engagement was not collected in the current sample. We do not believe this had any impact on differential reactivity across the two inductions for several reasons. First, all participants labeled as meeting diagnostic criteria, met such criteria at the time of the experiment (regardless of current treatment status). Second, the goal here was to compare two procedures within which all participants who met clinical levels of GAD or depression were block randomized. Thus, factors such as treatment status or medication use were likely distributed similarly between compared conditions and therefore unlikely to have had any effect on the differential reactivity found here.

In summary, our findings suggest that personalized induction methods are both effective and ecologically valid tools for eliciting repetitive negative thinking in experimental settings. For ease of reference, we propose collectively labeling these procedures as Induction Methods for Personalized Repetitive Negative Thinking (IMPRINT). We hope that these findings, along with the IMPRINT framework, will guide future research in selecting valid induction procedures and contribute to a deeper understanding of the mechanisms underlying worry and rumination.

## Supplementary Material

supplement

## Figures and Tables

**Table 1:** Demographic characteristics.

Variable	Total (*N* = 355)	GAD (*n* = 118)	Depression (*n* = 113)	LS (*n* = 124)	Statistics			
Categorical variables					*df*	χ^2^	*p*	*V*
Gender, *n* (%)					2	2.16	.340	.08
Women	282 (79.4)	99 (83.9)	87 (77.0)	96 (77.4)				
Men	73 (20.6)	19 (16.1)	26 (23.0)	28 (22.6)				
Race, *n* (%)					8	9.08	.336	.11
White	267 (75.2)	93 (75.0)	81 (65.3)	93 (75.0)				
Asian	37 (10.4)	13 (10.5)	13 (10.5)	11 (8.9)				
Hispanic	22 (6.2)	6 (4.8)	9 (7.3)	7 (5.6)				
Black	18 (5.1)	1 (0.8)	8 (6.5)	9 (7.3)				
Others	11 (3.1)	5 (4.0)	2 (1.6)	4 (3.2)				
Continuous variables					*df*	*F*	*p*	*η* ^*2*^
Age, *M* (*SD*)	18.23 (2.83)	18.30 (3.21)	18.34 (2.85)	18.06 (2.42)	2, 228.60	.377	.687	.00
GAD-Q-IV, *M* (*SD*)	4.88 (3.88)	9.10 (1.58)	4.40 (3.10)	1.30 (1.35)	2, 213.70	846.47	< .001	.89
BDI-2, *M* (*SD*)	12.35 (10.42)	8.92 (2.93)	25.75 (6.19)	3.39 (3.50)	2, 217.65	570.8	< .001	.84

*Note*. GAD = Generalized Anxiety Disorder; LS = low anxiety/depression symptom; GAD-Q-IV = Generalized Anxiety Disorder Questionnaire-IV-Continuous Score; BDI-2 = Beck Depression Inventory-2.

**Table 2:** Means and standard deviations of self-reported worry and rumination from baseline to induction, by induction method (personalized vs. scripted) and induction focus (worry vs. rumination) (*N* = 355).

Variables	Personalized method (*n* = 180)				Scripted method (*n* = 175)			
	Worry induction (*n* = 90)		Rumination induction (*n* = 90)		Worry induction (*n* = 89)		Rumination induction (*n* = 86)	
	Baseline	Induction	Baseline	Induction	Baseline	Induction	Baseline	Induction
Self-reported worry, *M* (*SD*)	.79 (1.27)	5.14 (1.44)	1.26 (1.71)	3.03 (2.12)	1.03 (1.51)	3.97 (2.11)	1.07 (1.52)	2.12 (1.95)
Self-reported rumination, *M*(*SD*)	.70 (1.20)	2.94 (2.34)	1.33 (1.86)	5.72 (1.82)	.90 (1.37)	2.84 (2.30)	1.17 (1.64)	2.69 (2.40)

*Note*. Worry and rumination scores were rated on the 9-point Likert scale ranging from 0 to 8.

## Data Availability

Data will be made available on request

## Footnotes

3 Table S1 presents the sample sizes for each cell of the 2 × 2 × 3 between-subjects design.

4 Continuous items of the GAD-Q-IV were used to calculate Cronbach’s α.

## Appendix A. Supporting information

Supplementary data associated with this article can be found in the online version at doi:10.1016/j.janxdis.2026.103148.
