# Supplementary material for: Development and validation of experimental induction tasks for worry and rumination: A comparison of personalized and scripted approaches
Source: J Anxiety Disord. Author manuscript; Available in PMC 2026 Apr 30. (PMC13131129; doi:10.1016/j.janxdis.2026.103148)
Supplement: supplement [file NIHMS2161747-supplement-supplement.docx]

**Supplementary Materials**

**Table S1. Participant distribution across induction method, induction focus, and group in the 2×2×3 between-subject design (*N* = 355)**

| Group | Personalized (*n* = 180) | | Scripted (*n* = 175) | |
| --- | --- | --- | --- | --- |
|  | Worry induction  (*n* = 90) | Rumination induction  (*n* = 90) | Worry induction  (*n* = 89) | Rumination induction  (*n* = 86) |
| GAD (*n* = 117) | 30 | 32 | 28 | 28 |
| Depression (*n* = 113) | 27 | 28 | 30 | 28 |
| LS (*n* = 124) | 33 | 30 | 31 | 30 |

*Note*. GAD = Generalized Anxiety Disorder; LS = low anxiety/depression symptom

**Table S2. Effects of induction method (personalized vs. scripted worry inductions) and time on self-reported worry and rumination (addressing RQ1).**

| Fixed effect | Self-reported worry (*n* = 173) | | | | | Self-reported rumination (*n* = 172) | | | | |
| --- | --- | --- | --- | --- | --- | --- | --- | --- | --- | --- |
|  | *b* | *SE* | *df* | *t* | *p* | *b* | *SE* | *df* | *t* | *p* |
| Intercept | 1.01 | .17 | 341.68 | 5.95^***^ | < .001 | 1.18 | .21 | 318.96 | 5.66^***^ | < .001 |
| Induction method | -.21 | .24 | 350.47 | -.88 | .381 | .17 | .29 | 331.67 | .59 | .555 |
| Time | 2.93 | .21 | 181.68 | 13.76^***^ | < .001 | 1.51 | .24 | 176.87 | 6.29^***^ | < .001 |
| Induction method × time | 1.42 | .30 | 181.68 | 4.73^***^ | < .001 | 2.88 | .34 | 176.87 | 8.56^***^ | < .001 |

*Note*. ^*^ *p* < .05, ^**^ *p* < .01, ^***^ *p* < .001.

**Table S3. Effects of induction focus (worry vs. rumination) and time on self-reported worry and rumination within the personalized induction condition (addressing RQ2).**

| Fixed effect | Self-reported worry (*n* = 180) | | | | | Self-reported rumination (*n* = 180) | | | | |
| --- | --- | --- | --- | --- | --- | --- | --- | --- | --- | --- |
|  | *b* | *SE* | *df* | *t* | *p* | *b* | *SE* | *df* | *t* | *p* |
| Intercept | .79 | .18 | 344.32 | 4.49^***^ | < .001 | .70 | .19 | 345.56 | 3.59^***^ | < .001 |
| Induction focus | .47 | .25 | 344.32 | 1.88 | .061 | .63 | .28 | 345.56 | 2.30^*^ | .022 |
| Time | 4.36 | .22 | 178.00 | 19.42^***^ | < .001 | 2.24 | .25 | 178.00 | 8.96^***^ | < .001 |
| Induction focus × time | -2.58 | .32 | 178.00 | -8.13^***^ | < .001 | 2.14 | .35 | 178.00 | 6.05^***^ | < .001 |

*Note*. ^*^ *p* < .05, ^**^ *p* < .01, ^***^ *p* < .001.

**Table S4. Effects of induction focus (worry vs. rumination) and time on self-reported worry and rumination within the scripted induction condition (addressing RQ2).**

| Fixed effect | Self-reported worry (*n* = 175) | | | | | Self-reported rumination (*n* = 175) | | | | |
| --- | --- | --- | --- | --- | --- | --- | --- | --- | --- | --- |
|  | *b* | *SE* | *df* | *t* | *p* | *b* | *SE* | *df* | *t* | *p* |
| Intercept | 1.03 | .19 | 300.13 | 5.44^***^ | < .001 | .90 | .21 | 278.57 | 4.30^***^ | < .001 |
| Induction focus | .04 | .27 | 300.13 | .13 | .894 | .28 | .30 | 278.57 | .92 | .356 |
| Time | 2.93 | .21 | 173.00 | 13.98^***^ | < .001 | 1.94 | .21 | 173.00 | 9.22^***^ | < .001 |
| Induction focus × time | -1.89 | .30 | 173.00 | -6.30^***^ | < .001 | -.43 | .30 | 173.00 | -1.44 | .152 |

*Note*. ^*^ *p* < .05, ^**^ *p* < .01, ^***^ *p* < .001.

**Table S5. Effects of induction method (personalized vs. scripted) and time on self-reported worry and rumination, with group as a factor (addressing RQ3).**

| Fixed effect | Self-reported worry (*n* = 173) | | | | | Self-reported rumination (*n* = 172) | | | | |
| --- | --- | --- | --- | --- | --- | --- | --- | --- | --- | --- |
|  | *b* | *SE* | *df* | *t* | *p* | *b* | *SE* | *df* | *t* | *p* |
| Intercept | .52 | .28 | 333.20 | 1.85 | .066 | .70 | .35 | 306.40 | 2.01^*^ | .045 |
| Group |  |  |  |  |  |  |  |  |  |  |
| GAD vs. LS | .81 | .40 | 338.66 | 2.00^*^ | .046 | .79 | .50 | 313.50 | 1.58 | .115 |
| Depression vs. LS | .76 | .40 | 334.51 | 1.90 | .059 | .70 | .50 | 313.40 | 1.39 | .165 |
| Induction method | -.33 | .39 | 333.20 | -.86 | .391 | .00 | .49 | 306.40 | .00 | 1.000 |
| Time | 2.58 | .36 | 178.86 | 7.23^***^ | .000 | .97 | .41 | 174.10 | 2.38^*^ | .018 |
| Two-way interactions |  |  |  |  |  |  |  |  |  |  |
| GAD × Induction method | .36 | .56 | 345.36 | .64 | .524 | .20 | .69 | 321.30 | .29 | .775 |
| Depression × Induction method | .02 | .57 | 337.21 | .04 | .967 | .30 | .70 | 322.60 | .43 | .670 |
| GAD × Time | .13 | .52 | 178.86 | .26 | .797 | .93 | .58 | 174.10 | 1.59 | .115 |
| Depression × Time | .92 | .51 | 178.86 | 1.81 | .073 | .75 | .58 | 174.10 | 1.28 | .203 |
| Induction method × Time | 2.15 | .50 | 178.86 | 4.32^***^ | .000 | 3.87 | .57 | 174.10 | 6.73^***^ | .000 |
| Three-way interactions |  |  |  |  |  |  |  |  |  |  |
| GAD × Induction method × Time | -.63 | .72 | 178.86 | -.87 | .386 | -1.70 | .81 | 174.10 | -2.09^*^ | .038 |
| Depression × Induction method × Time | -1.61 | .72 | 178.86 | -2.22^*^ | .028 | -1.30 | .83 | 174.10 | -1.57 | .119 |

*Note*. GAD = Generalized Anxiety Disorder; LS = low anxiety/depression symptom. ^*^ *p* < .05, ^**^ *p* < .01, ^***^ *p* < .001.

**Table S6. Effects of induction focus (worry vs. rumination) and time on self-reported worry and rumination within the personalized induction condition, with group as a factor (addressing RQ3).**

| Fixed effect | Self-reported worry (*n* = 180) | | | | | Self-reported rumination (*n* = 180) | | | | |
| --- | --- | --- | --- | --- | --- | --- | --- | --- | --- | --- |
|  | *b* | *SE* | *df* | *t* | *p* | *b* | *SE* | *df* | *t* | *p* |
| Intercept | .18 | .28 | 342.23 | .65 | .518 | .18 | .31 | 340.94 | .58 | .564 |
| Group |  |  |  |  |  |  |  |  |  |  |
| GAD vs. LS | 1.12 | .41 | 342.23 | 2.75^**^ | .006 | 1.12 | .46 | 340.94 | 2.45^*^ | .015 |
| Depression vs. LS | .78 | .42 | 342.23 | 1.87 | .063 | .48 | .47 | 340.94 | 1.03 | .302 |
| Induction focus | .15 | .41 | 342.23 | .37 | .710 | .52 | .46 | 340.94 | 1.14 | .256 |
| Time | 4.73 | .37 | 174.00 | 12.77^***^ | < .001 | 1.91 | .41 | 174.00 | 4.64^***^ | < .001 |
| Two-way interactions |  |  |  |  |  |  |  |  |  |  |
| GAD × Induction focus | .36 | .58 | 342.23 | .63 | .532 | -.19 | .65 | 340.94 | -.30 | .765 |
| Depression × Induction focus | .49 | .60 | 342.23 | .83 | .409 | .49 | .67 | 340.94 | .74 | .460 |
| GAD × Time | -.49 | .54 | 174.00 | -.92 | .359 | -.01 | .60 | 174.00 | -.02 | .988 |
| Depression × Time | -.69 | .55 | 174.00 | -1.25 | .213 | 1.13 | .61 | 174.00 | 1.84 | .068 |
| Induction focus × Time | -2.59 | .54 | 174.00 | -4.84^***^ | < .001 | 2.92 | .60 | 174.00 | 4.90^***^ | < .001 |
| Three-way interactions |  |  |  |  |  |  |  |  |  |  |
| GAD × Induction focus × Time | -.33 | .76 | 174.00 | -.43 | .668 | -.76 | .85 | 174.00 | -.90 | .369 |
| Depression × Induction focus × Time | .49 | .79 | 174.00 | .62 | .537 | -1.68 | .87 | 174.00 | -1.92^*^ | .057 |

*Note*. GAD = Generalized Anxiety Disorder; LS = low anxiety/depression symptom. ^*^ *p* < .05, ^**^ *p* < .01, ^***^ *p* < .001.

**Table S7. Effects of induction focus (worry vs. rumination) and time on self-reported worry and rumination within the scripted induction condition, with group as a factor (addressing RQ3).**

| Fixed effect | Self-reported worry (*n* = 175) | | | | | Self-reported rumination (*n* = 175) | | | | |
| --- | --- | --- | --- | --- | --- | --- | --- | --- | --- | --- |
|  | *b* | *SE* | *df* | *t* | *p* | *b* | *SE* | *df* | *t* | *p* |
| Intercept | .52 | .30 | 304.06 | 1.70 | .090 | .39 | .33 | 281.25 | 1.16 | .248 |
| Group |  |  |  |  |  |  |  |  |  |  |
| GAD vs. LS | .84 | .44 | 304.06 | 1.91 | .057 | .36 | .49 | 281.25 | .75 | .456 |
| Depression vs. LS | .75 | .43 | 304.06 | 1.74 | .084 | 1.18 | .48 | 281.25 | 2.47^*^ | .014 |
| Induction focus | -.15 | .43 | 304.06 | -.35 | .730 | .31 | .48 | 281.25 | .66 | .512 |
| Time | 2.58 | .35 | 169.00 | 7.38^***^ | < .001 | 1.48 | .35 | 169.00 | 4.23^***^ | < .001 |
| Two-way interactions |  |  |  |  |  |  |  |  |  |  |
| GAD × Induction focus | .19 | .62 | 304.06 | .30 | .767 | .44 | .69 | 281.25 | .63 | .527 |
| Depression × Induction focus | .38 | .62 | 304.06 | .62 | .537 | -.52 | .68 | 281.25 | -.76 | .445 |
| GAD × Time | .13 | .51 | 169.00 | .26 | .793 | .19 | .51 | 169.00 | .38 | .703 |
| Depression × Time | .92 | .50 | 169.00 | 1.84 | .067 | 1.18 | .50 | 169.00 | 2.36^*^ | .019 |
| Induction focus × Time | -1.98 | .50 | 169.00 | -3.97^***^ | < .001 | -.52 | .50 | 169.00 | -1.03 | .303 |
| Three-way interactions |  |  |  |  |  |  |  |  |  |  |
| GAD × Induction focus × Time | 1.02 | .72 | 169.00 | 1.41 | .161 | .73 | .72 | 169.00 | 1.01 | .314 |
| Depression × Induction focus × Time | -.70 | .71 | 169.00 | -.98 | .330 | -.44 | .72 | 169.00 | -.61 | .545 |

*Note*. GAD = Generalized Anxiety Disorder; LS = low anxiety/depression symptom. ^*^ *p* < .05, ^**^ *p* < .01, ^***^ *p* < .001.

**Table S8. Demographically adjusted effects of induction method (personalized vs. scripted worry inductions) and time on self-reported worry and rumination (Sensitivity Analysis 1).**

| Fixed effect | Self-reported worry (*n* = 173) | | | | | Self-reported rumination (*n* = 172) | | | | |
| --- | --- | --- | --- | --- | --- | --- | --- | --- | --- | --- |
|  | *b* | *SE* | *df* | *t* | *p* | *b* | *SE* | *df* | *t* | *p* |
| Intercept | .88 | .78 | 272.37 | 1.14 | .256 | .67 | .69 | 175.54 | .96 | .337 |
| Induction method | -.23 | .24 | 340.62 | -.94 | .350 | .13 | .30 | 320.16 | .42 | .672 |
| Time | 2.93 | .21 | 179.44 | 13.77^***^ | < .001 | 1.51 | .24 | 177.15 | 6.29^***^ | < .001 |
| Age | .01 | .04 | 263.93 | .30 | .765 | .03 | .04 | 166.27 | .88 | .381 |
| Gender | -.56 | .24 | 158.56 | -2.33^*^ | .021 | -.46 | .30 | 165.09 | -1.53 | .128 |
| Race |  |  |  |  |  |  |  |  |  |  |
| Asian vs. White | -.11 | .36 | 167.75 | -.30 | .763 | .32 | .37 | 165.59 | .86 | .390 |
| Hispanic vs. White | .07 | .38 | 167.17 | .18 | .860 | .04 | .53 | 165.55 | .07 | .947 |
| Black vs. White | .44 | .42 | 167.15 | 1.05 | .295 | -.20 | .59 | 165.72 | -.34 | .734 |
| Others vs. White | -.05 | .64 | 167.27 | -.08 | .933 | .05 | .63 | 166.00 | .08 | .934 |
| Induction method × Time | 1.42 | .30 | 179.44 | 4.74^***^ | < .001 | 2.88 | .34 | 177.15 | 8.56^***^ | < .001 |

*Note*. ^*^ *p* < .05, ^**^ *p* < .01, ^***^ *p* < .001.

**Table S9. Demographically adjusted effects of induction focus (worry vs. rumination) and time on self-reported worry and rumination within the personalized induction condition (Sensitivity Analysis 1).**

| Fixed effect | Self-reported worry (*n* = 180) | | | | | Self-reported rumination (*n* = 180) | | | | |
| --- | --- | --- | --- | --- | --- | --- | --- | --- | --- | --- |
|  | *b* | *SE* | *df* | *t* | *p* | *b* | *SE* | *df* | *t* | *p* |
| Intercept | -1.49 | 1.51 | 173.92 | -.99 | .324 | -.37 | 1.71 | 173.86 | -.22 | .828 |
| Induction focus | .49 | .25 | 337.05 | 1.97^*^ | .049 | .62 | .28 | 334.99 | 2.22^*^ | .027 |
| Time | 4.36 | .22 | 178.00 | 19.42^***^ | < .001 | 2.24 | .25 | 178.00 | 8.96^***^ | < .001 |
| Age | .12 | .08 | 172.00 | 1.52 | .131 | .06 | .09 | 172.00 | .63 | .529 |
| Gender | -.63 | .25 | 172.00 | -2.47^*^ | .015 | -.28 | .29 | 172.00 | -.98 | .330 |
| Race |  |  |  |  |  |  |  |  |  |  |
| Asian vs. White | .44 | .33 | 172.00 | 1.32 | .188 | .34 | .38 | 172.00 | .88 | .378 |
| Hispanic vs. White | .58 | .36 | 172.00 | 1.64 | .104 | .18 | .40 | 172.00 | .44 | .664 |
| Black vs. White | .65 | .53 | 172.00 | 1.23 | .222 | -.11 | .60 | 172.00 | -.19 | .850 |
| Others vs. White | -.25 | .46 | 172.00 | -.55 | .585 | .24 | .53 | 172.00 | .46 | .649 |
| Induction focus × Time | -2.58 | .32 | 178.00 | -8.13^***^ | < .001 | 2.14 | .35 | 178.00 | 6.05^***^ | < .001 |

*Note*. ^*^ *p* < .05, ^**^ *p* < .01, ^***^ *p* < .001.

**Table S10. Demographically adjusted effects of induction focus (worry vs. rumination) and time on self-reported worry and rumination within the scripted induction condition (Sensitivity Analysis 1).**

| Fixed effect | Self-reported worry (*n* = 175) | | | | | Self-reported rumination (*n* = 175) | | | | |
| --- | --- | --- | --- | --- | --- | --- | --- | --- | --- | --- |
|  | *b* | *SE* | *df* | *t* | *p* | *b* | *SE* | *df* | *t* | *p* |
| Intercept | .90 | .59 | 177.85 | 1.53 | .128 | .63 | .67 | 175.49 | .94 | .347 |
| Induction focus | .05 | .28 | 287.42 | .19 | .846 | .31 | .30 | 266.61 | 1.02 | .308 |
| Time | 2.93 | .21 | 173.00 | 13.98^***^ | < .001 | 1.94 | .21 | 173.00 | 9.22^***^ | < .001 |
| Age | .01 | .03 | 167.00 | .45 | .655 | .02 | .03 | 167.00 | .57 | .571 |
| Gender | -.52 | .27 | 167.00 | -1.89 | .060 | -.48 | .31 | 167.00 | -1.56 | .121 |
| Race |  |  |  |  |  |  |  |  |  |  |
| Asian vs. White | .08 | .36 | 167.00 | .23 | .820 | .16 | .41 | 167.00 | .39 | .696 |
| Hispanic vs. White | -.40 | .55 | 167.00 | -.74 | .464 | -.67 | .63 | 167.00 | -1.06 | .289 |
| Black vs. White | .08 | .45 | 167.00 | .19 | .853 | -.01 | .52 | 167.00 | -.03 | .980 |
| Others vs. White | -.16 | .88 | 167.00 | -.18 | .858 | 1.16 | 1.00 | 167.00 | 1.16 | .248 |
| Induction focus × Time | -1.89 | .30 | 173.00 | -6.30^***^ | < .001 | -.43 | .30 | 173.00 | -1.44 | .152 |

*Note*. ^*^ *p* < .05, ^**^ *p* < .01, ^***^ *p* < .001.

**Table S11. Demographically adjusted effects of induction method (personalized vs. scripted) and time on self-reported worry and rumination, with group as a factor (Sensitivity Analysis 1).**

| Fixed effect | Self-reported worry (*n* = 173) | | | | | Self-reported rumination (*n* = 172) | | | | |
| --- | --- | --- | --- | --- | --- | --- | --- | --- | --- | --- |
|  | *b* | *SE* | *df* | *t* | *p* | *b* | *SE* | *df* | *t* | *p* |
| Intercept | .51 | .79 | 249.17 | .64 | .524 | .33 | .73 | 190.71 | .46 | .650 |
| Group |  |  |  |  |  |  |  |  |  |  |
| GAD vs. LS | .86 | .41 | 330.03 | 2.13^*^ | .034 | .74 | .51 | 301.36 | 1.46 | .145 |
| Depression vs. LS | .63 | .40 | 324.44 | 1.56 | .119 | .65 | .51 | 302.41 | 1.29 | .198 |
| Induction method | -.44 | .39 | 324.09 | -1.11 | .267 | -.03 | .50 | 292.36 | -.06 | .952 |
| Time | 2.58 | .36 | 176.47 | 7.22^***^ | < .001 | .97 | .41 | 174.20 | 2.38^*^ | .018 |
| Age | .01 | .04 | 238.65 | .18 | .861 | .03 | .04 | 163.68 | .68 | .495 |
| Gender | -.52 | .23 | 154.25 | -2.24^*^ | .027 | -.36 | .30 | 162.66 | -1.21 | .228 |
| Race |  |  |  |  |  |  |  |  |  |  |
| Asian vs. White | -.17 | .34 | 164.05 | -.49 | .626 | .29 | .37 | 162.76 | .80 | .424 |
| Hispanic vs. White | .07 | .37 | 165.83 | .19 | .848 | -.05 | .52 | 163.01 | -.10 | .924 |
| Black vs. White | .49 | .40 | 165.96 | 1.21 | .226 | -.05 | .58 | 164.09 | -.08 | .936 |
| Others vs. White | -.26 | .61 | 166.03 | -.43 | .671 | .09 | .62 | 163.20 | .15 | .880 |
| Two-way interactions |  |  |  |  |  |  |  |  |  |  |
| GAD × Induction method | .38 | .56 | 337.55 | .68 | .499 | .19 | .70 | 311.03 | .27 | .788 |
| Depression × Induction method | .28 | .58 | 325.73 | .48 | .631 | .31 | .71 | 312.24 | .43 | .665 |
| GAD × Time | .13 | .52 | 176.47 | .26 | .797 | .93 | .58 | 174.20 | 1.58 | .115 |
| Depression × Time | .92 | .51 | 176.47 | 1.80 | .073 | .75 | .58 | 174.20 | 1.28 | .203 |
| Induction method × Time | 2.15 | .50 | 176.47 | 4.31^***^ | < .001 | 3.87 | .57 | 174.20 | 6.73^***^ | < .001 |
| Three-way interactions |  |  |  |  |  |  |  |  |  |  |
| GAD × Induction method × Time | -.63 | .72 | 176.47 | -.87 | .386 | -1.70 | .81 | 174.20 | -2.09^*^ | .038 |
| Depression × Induction method × Time | -1.61 | .73 | 176.47 | -2.22^*^ | .028 | -1.30 | .83 | 174.20 | -1.57 | .119 |

*Note*. GAD = Generalized Anxiety Disorder; LS = low anxiety/depression symptom. ^*^ *p* < .05, ^**^ *p* < .01, ^***^ *p* < .001.

**Table S12. Demographically adjusted effects of induction focus (worry vs. rumination) and time on self-reported worry and rumination within the personalized induction condition, with group as a factor (Sensitivity Analysis 1).**

| Fixed effect | Self-reported worry (*n* = 180) | | | | | Self-reported rumination (*n* = 180) | | | | |
| --- | --- | --- | --- | --- | --- | --- | --- | --- | --- | --- |
|  | *b* | *SE* | *df* | *t* | *p* | *b* | *SE* | *df* | *t* | *p* |
| Intercept | -.62 | 1.45 | 173.54 | -.43 | .668 | .48 | 1.68 | 173.10 | .29 | .775 |
| Group |  |  |  |  |  |  |  |  |  |  |
| GAD vs. LS | 1.16 | .41 | 332.80 | 2.83^**^ | .005 | 1.13 | .47 | 327.94 | 2.41^*^ | .016 |
| Depression vs. LS | .89 | .42 | 333.21 | 2.10^*^ | .036 | .56 | .48 | 328.42 | 1.17 | .244 |
| Induction focus | .24 | .41 | 334.53 | .60 | .551 | .53 | .46 | 330.03 | 1.15 | .250 |
| Time | 4.73 | .37 | 174.00 | 12.77^***^ | < .001 | 1.91 | .41 | 174.00 | 4.64^***^ | < .001 |
| Age | .04 | .08 | 168.00 | .51 | .609 | -.02 | .09 | 168.00 | -.18 | .854 |
| Gender | -.52 | .25 | 168.00 | -2.13^*^ | .035 | -.27 | .28 | 168.00 | -.96 | .339 |
| Race |  |  |  |  |  |  |  |  |  |  |
| Asian vs. White | .27 | .32 | 168.00 | .85 | .396 | .17 | .37 | 168.00 | .46 | .647 |
| Hispanic vs. White | .52 | .34 | 168.00 | 1.54 | .125 | .05 | .39 | 168.00 | .12 | .904 |
| Black vs. White | 1.05 | .51 | 168.00 | 2.07^*^ | .040 | .33 | .59 | 168.00 | .56 | .576 |
| Others vs. White | -.25 | .44 | 168.00 | -.56 | .574 | .18 | .51 | 168.00 | .34 | .732 |
| Two-way interactions |  |  |  |  |  |  |  |  |  |  |
| GAD × Induction focus | .27 | .58 | 334.51 | .46 | .644 | -.21 | .66 | 330.00 | -.32 | .746 |
| Depression × Induction focus | .40 | .59 | 335.28 | .67 | .505 | .44 | .68 | 330.95 | .66 | .511 |
| GAD × Time | -.49 | .54 | 174.00 | -.92 | .359 | -.01 | .60 | 174.00 | -.02 | .988 |
| Depression × Time | -.69 | .55 | 174.00 | -1.25 | .213 | 1.13 | .61 | 174.00 | 1.84 | .068 |
| Induction focus × Time | -2.59 | .54 | 174.00 | -4.84^***^ | < .001 | 2.92 | .60 | 174.00 | 4.90^***^ | < .001 |
| Three-way interactions |  |  |  |  |  |  |  |  |  |  |
| GAD × Induction focus × Time | -.33 | .76 | 174.00 | -.43 | .668 | -.76 | .85 | 174.00 | -.90 | .369 |
| Depression × Induction focus × Time | .49 | .79 | 174.00 | .62 | .537 | -1.68 | .87 | 174.00 | -1.92 | .057 |

*Note*. GAD = Generalized Anxiety Disorder; LS = low anxiety/depression symptom. ^*^ *p* < .05, ^**^ *p* < .01, ^***^ *p* < .001.

**Table S13. Demographically adjusted effects of induction focus (worry vs. rumination) and time on self-reported worry and rumination within the scripted induction condition, with group as a factor (Sensitivity Analysis 1).**

| Fixed effect | Self-reported worry (*n* = 175) | | | | | Self-reported rumination (*n* = 175) | | | | |
| --- | --- | --- | --- | --- | --- | --- | --- | --- | --- | --- |
|  | *b* | *SE* | *df* | *t* | *p* | *b* | *SE* | *df* | *t* | *p* |
| Intercept | .36 | .61 | 191.75 | .59 | .554 | .06 | .69 | 185.17 | .08 | .933 |
| Group |  |  |  |  |  |  |  |  |  |  |
| GAD vs. LS | .82 | .45 | 292.09 | 1.83 | .068 | .33 | .49 | 270.03 | .67 | .504 |
| Depression vs. LS | .71 | .44 | 291.31 | 1.62 | .106 | 1.20 | .48 | 269.26 | 2.48^*^ | .014 |
| Induction focus | -.15 | .44 | 291.89 | -.34 | .731 | .34 | .48 | 269.84 | .70 | .484 |
| Time | 2.58 | .35 | 169.00 | 7.38^***^ | < .001 | 1.48 | .35 | 169.00 | 4.23^***^ | < .001 |
| Age | .01 | .03 | 163.00 | .50 | .621 | .02 | .03 | 163.00 | .67 | .504 |
| Gender | -.35 | .25 | 163.00 | -1.39 | .166 | -.27 | .29 | 163.00 | -.93 | .355 |
| Race |  |  |  |  |  |  |  |  |  |  |
| Asian vs. White | .16 | .34 | 163.00 | .48 | .633 | .32 | .39 | 163.00 | .82 | .414 |
| Hispanic vs. White | -.41 | .51 | 163.00 | -.79 | .430 | -.70 | .59 | 163.00 | -1.18 | .238 |
| Black vs. White | .06 | .43 | 163.00 | .15 | .882 | -.30 | .49 | 163.00 | -.60 | .549 |
| Others vs. White | -.34 | .82 | 163.00 | -.41 | .681 | .86 | .94 | 163.00 | .91 | .363 |
| Two-way interactions |  |  |  |  |  |  |  |  |  |  |
| GAD × Induction focus | .19 | .64 | 291.13 | .31 | .760 | .42 | .70 | 269.08 | .60 | .547 |
| Depression × Induction focus | .40 | .63 | 292.25 | .64 | .524 | -.54 | .69 | 270.19 | -.79 | .431 |
| GAD × Time | .13 | .51 | 169.00 | .26 | .793 | .19 | .51 | 169.00 | .38 | .703 |
| Depression × Time | .92 | .50 | 169.00 | 1.84 | .067 | 1.18 | .50 | 169.00 | 2.36^*^ | .019 |
| Induction focus × Time | -1.98 | .50 | 169.00 | -3.97^***^ | < .001 | -.52 | .50 | 169.00 | -1.03 | .303 |
| Three-way interactions |  |  |  |  |  |  |  |  |  |  |
| GAD × Induction focus × Time | 1.02 | .72 | 169.00 | 1.41 | .161 | .73 | .72 | 169.00 | 1.01 | .314 |
| Depression × Induction focus × Time | -.70 | .71 | 169.00 | -.98 | .330 | -.44 | .72 | 169.00 | -.61 | .545 |

*Note*. GAD = Generalized Anxiety Disorder; LS = low anxiety/depression symptom. ^*^ *p* < .05, ^**^ *p* < .01, ^***^ *p* < .001.

**Table S14. Effects of rumination induction method (personalized vs. scripted) and time on self-reported rumination in the depression group (Sensitivity Analysis 2).**

| Fixed effect | Self-reported rumination (*n* = 54) | | | | |
| --- | --- | --- | --- | --- | --- |
|  | *b* | *SE* | *df* | *t* | *p* |
| Intercept | 1.40 | .37 | 97.99 | 3.74^***^ | < .001 |
| Induction method | .29 | .52 | 104.71 | .56 | .577 |
| Time | 1.71 | .41 | 55.84 | 4.14^***^ | < .001 |
| Induction method × Time | 2.57 | .59 | 55.84 | 4.39^***^ | < .001 |

*Note*. ^*^ *p* < .05, ^**^ *p* < .01, ^***^ *p* < .001.

**Table S15. Effects of induction focus (worry vs. rumination) and time on self-reported rumination in the depression group under the scripted induction condition (Sensitivity Analysis 2).**

| Fixed effect | Self-reported rumination (*n* = 58) | | | | |
| --- | --- | --- | --- | --- | --- |
|  | *b* | *SE* | *df* | *t* | *p* |
| Intercept | 1.57 | .38 | 92.04 | 4.10^***^ | < .001 |
| Induction focus | -.21 | .55 | 92.04 | -.38 | .704 |
| Time | 2.67 | .40 | 56.00 | 6.75^***^ | < .001 |
| Induction focus × Time | -.95 | .57 | 56.00 | -1.67 | .100 |

*Note*. ^*^ *p* < .05, ^**^ *p* < .01, ^***^ *p* < .001.

**Table S16. Effects of induction method (personalized vs. scripted) and time on self-reported worry and rumination, with GAD-Q-IV score as a factor (addressing RQ3).**

| Fixed effect | Self-reported worry (*n* = 173) | | | | | Self-reported rumination (*n* = 172) | | | | |
| --- | --- | --- | --- | --- | --- | --- | --- | --- | --- | --- |
|  | *b* | *SE* | *df* | *t* | *p* | *b* | *SE* | *df* | *t* | *p* |
| Intercept | .92 | .25 | 334.48 | 3.67^***^ | < .001 | .84 | .35 | 309.44 | 2.39^*^ | .017 |
| GAD-Q-IV | .02 | .04 | 340.54 | .56 | .577 | .06 | .05 | 310.35 | 1.20 | .233 |
| Induction method | -.74 | .37 | 336.90 | -2.00^*^ | .046 | -.06 | .49 | 312.96 | -.11 | .910 |
| Time | 2.80 | .32 | 179.58 | 8.88^***^ | < .001 | .87 | .41 | 175.45 | 2.15^*^ | .033 |
| GAD-Q-IV × Induction method | .11 | .06 | 348.39 | 1.72 | .086 | .05 | .07 | 315.25 | .61 | .543 |
| GAD-Q-IV × Time | .03 | .06 | 179.58 | .56 | .576 | .12 | .06 | 175.45 | 1.94 | .055 |
| Induction method × Time | 1.84 | .46 | 179.58 | 3.96^***^ | < .001 | 4.01 | .56 | 175.45 | 7.13^***^ | < .001 |
| GAD-Q-IV × Induction method × Time | -.09 | .08 | 179.58 | -1.15 | .251 | -.21 | .09 | 175.45 | -2.49^*^ | .014 |

*Note*. GAD-Q-IV = Generalized Anxiety Disorder Questionnaire–IV total score. ^*^ *p* < .05, ^**^ *p* < .01, ^***^ *p* < .001.

**Table S17. Effects of induction focus (worry vs. rumination) and time on self-reported worry and rumination within the personalized induction condition, with GAD-Q-IV score as a factor (addressing RQ3).**

| Fixed effect | Self-reported worry (*n* = 180) | | | | | Self-reported rumination (*n* = 180) | | | | |
| --- | --- | --- | --- | --- | --- | --- | --- | --- | --- | --- |
|  | *b* | *SE* | *df* | *t* | *p* | *b* | *SE* | *df* | *t* | *p* |
| Intercept | .19 | .27 | 346.71 | .71 | .475 | .18 | .31 | 345.37 | .59 | .553 |
| GAD-Q-IV | .13 | .04 | 346.71 | 2.79^**^ | .006 | .11 | .05 | 345.37 | 2.17^*^ | .031 |
| Induction focus | .25 | .39 | 346.71 | .64 | .521 | .60 | .44 | 345.37 | 1.36 | .173 |
| Time | 4.64 | .36 | 176.00 | 12.86^***^ | < .001 | 1.93 | .40 | 176.00 | 4.80^***^ | < .001 |
| GAD-Q-IV × Induction focus | .03 | .06 | 346.71 | .46 | .645 | .00 | .07 | 345.37 | -.06 | .950 |
| GAD-Q-IV × Time | -.06 | .06 | 176.00 | -1.01 | .316 | .07 | .07 | 176.00 | 1.02 | .310 |
| Induction focus × Time | -2.65 | .52 | 176.00 | -5.09^***^ | < .001 | 2.96 | .58 | 176.00 | 5.11^***^ | < .001 |
| GAD-Q-IV × Induction focus × Time | .02 | .08 | 176.00 | .24 | .814 | -.16 | .09 | 176.00 | -1.76 | .081 |

*Note*. GAD-Q-IV = Generalized Anxiety Disorder Questionnaire–IV total score. ^*^ *p* < .05, ^**^ *p* < .01, ^***^ *p* < .001.

**Table S18. Effects of induction focus (worry vs. rumination) and time on self-reported worry and rumination within the scripted induction condition, with GAD-Q-IV score as a factor (addressing RQ3).**

| Fixed effect | Self-reported worry (*n* = 175) | | | | | Self-reported rumination (*n* = 175) | | | | |
| --- | --- | --- | --- | --- | --- | --- | --- | --- | --- | --- |
|  | *b* | *SE* | *df* | *t* | *p* | *b* | *SE* | *df* | *t* | *p* |
| Intercept | .91 | .27 | 299.26 | 3.33^***^ | < .001 | .90 | .31 | 276.40 | 2.92^**^ | .004 |
| GAD-Q-IV | .03 | .05 | 299.26 | .60 | .547 | .00 | .05 | 276.40 | .02 | .986 |
| Induction focus | -.20 | .42 | 299.26 | -.47 | .636 | -.06 | .47 | 276.40 | -.13 | .894 |
| Time | 2.80 | .31 | 171.00 | 9.17^***^ | < .001 | 1.91 | .31 | 171.00 | 6.14^***^ | < .001 |
| GAD-Q-IV × Induction focus | .04 | .07 | 299.26 | .55 | .580 | .06 | .08 | 276.40 | .82 | .411 |
| GAD-Q-IV × Time | .03 | .05 | 171.00 | .58 | .564 | .01 | .05 | 171.00 | .17 | .869 |
| Induction focus × Time | -2.58 | .47 | 171.00 | -5.48^***^ | < .001 | -1.03 | .48 | 171.00 | -2.16^*^ | .032 |
| GAD-Q-IV × Induction focus × Time | .12 | .08 | 171.00 | 1.61 | .109 | .11 | .08 | 171.00 | 1.42 | .157 |

*Note*. GAD-Q-IV = Generalized Anxiety Disorder Questionnaire–IV total score. ^*^ *p* < .05, ^**^ *p* < .01, ^***^ *p* < .001.

**Table S19. Effects of induction method (personalized vs. scripted) and time on self-reported worry and rumination, with BDI-2 score as a factor (addressing RQ3).**

| Fixed effect | Self-reported worry (*n* = 173) | | | | | Self-reported rumination (*n* = 172) | | | | |
| --- | --- | --- | --- | --- | --- | --- | --- | --- | --- | --- |
|  | *b* | *SE* | *df* | *t* | *p* | *b* | *SE* | *df* | *t* | *p* |
| Intercept | .71 | .24 | 338.27 | 2.96^**^ | .003 | .84 | .32 | 311.14 | 2.61^**^ | .009 |
| BDI-2 | .02 | .01 | 336.82 | 1.74 | .083 | .03 | .02 | 313.04 | 1.39 | .166 |
| Induction method | -.09 | .36 | 344.54 | -.25 | .800 | .03 | .46 | 314.90 | .06 | .951 |
| Time | 2.45 | .30 | 180.45 | 8.03^***^ | < .001 | 1.26 | .37 | 175.45 | 3.38^***^ | < .001 |
| BDI-2 × Induction method | -.01 | .02 | 342.59 | -.43 | .671 | .01 | .03 | 317.55 | .29 | .772 |
| BDI-2 × Time | .04 | .02 | 180.45 | 2.21^*^ | .028 | .02 | .02 | 175.45 | .86 | .391 |
| Induction method × Time | 2.17 | .45 | 180.45 | 4.80^***^ | < .001 | 3.23 | .54 | 175.45 | 6.02^***^ | < .001 |
| BDI-2 × Induction method × Time | -.06 | .03 | 180.45 | -2.18^*^ | .031 | -.03 | .03 | 175.45 | -.87 | .386 |

*Note*. BDI-2 = Beck Depression Inventory total score. ^*^ *p* < .05, ^**^ *p* < .01, ^***^ *p* < .001.

**Table S20. Effects of induction focus (worry vs. rumination) and time on self-reported worry and rumination within the personalized induction condition, with BDI-2 score as a factor (addressing RQ3).**

| Fixed effect | Self-reported worry (*n* = 180) | | | | | Self-reported rumination (*n* = 180) | | | | |
| --- | --- | --- | --- | --- | --- | --- | --- | --- | --- | --- |
|  | *b* | *SE* | *df* | *t* | *p* | *b* | *SE* | *df* | *t* | *p* |
| Intercept | .60 | .28 | 343.17 | 2.16^*^ | .031 | .71 | .31 | 342.75 | 2.31^*^ | .022 |
| BDI-2 | .02 | .02 | 343.17 | .86 | .393 | .00 | .02 | 342.75 | -.06 | .952 |
| Induction focus | -.02 | .40 | 343.17 | -.05 | .960 | .13 | .44 | 342.75 | .30 | .763 |
| Time | 4.62 | .36 | 176.00 | 12.81^***^ | < .001 | 1.61 | .40 | 176.00 | 4.02^***^ | < .001 |
| BDI-2 × Induction focus | .03 | .02 | 343.17 | 1.40 | .164 | .04 | .03 | 342.75 | 1.36 | .175 |
| BDI-2 × Time | -.02 | .02 | 176.00 | -.94 | .350 | .05 | .03 | 176.00 | 2.04^*^ | .043 |
| Induction focus × Time | -2.60 | .51 | 176.00 | -5.05^***^ | < .001 | 2.89 | .57 | 176.00 | 5.07^***^ | < .001 |
| BDI-2 × Induction focus × Time | .00 | .03 | 176.00 | .12 | .902 | -.06 | .04 | 176.00 | -1.73 | .085 |

*Note*. BDI-2 = Beck Depression Inventory total score. ^*^ *p* < .05, ^**^ *p* < .01, ^***^ *p* < .001.

**Table S21. Effects of induction focus (worry vs. rumination) and time on self-reported worry and rumination within the scripted induction condition, with BDI-2 score as a factor (addressing RQ3).**

| Fixed effect | Self-reported worry (*n* = 175) | | | | | Self-reported rumination (*n* = 175) | | | | |
| --- | --- | --- | --- | --- | --- | --- | --- | --- | --- | --- |
|  | *b* | *SE* | *df* | *t* | *p* | *b* | *SE* | *df* | *t* | *p* |
| Intercept | .74 | .27 | 303.33 | 2.78^**^ | .006 | .41 | .28 | 282.10 | 1.45 | .149 |
| BDI-2 | .02 | .02 | 303.33 | 1.53 | .126 | .04 | .02 | 282.10 | 2.36^*^ | .019 |
| Induction focus | -.13 | .39 | 303.33 | -.34 | .732 | .44 | .42 | 282.10 | 1.03 | .303 |
| Time | 2.45 | .30 | 171.00 | 8.12^***^ | < .001 | 1.18 | .30 | 171.00 | 3.99^***^ | < .001 |
| BDI-2 × Induction focus | .01 | .02 | 303.33 | .59 | .553 | -.01 | .03 | 282.10 | -.50 | .621 |
| BDI-2 × Time | .04 | .02 | 171.00 | 2.24^*^ | .027 | .06 | .02 | 171.00 | 3.57^***^ | < .001 |
| Induction focus × Time | -1.50 | .45 | 171.00 | -3.38^***^ | < .001 | .08 | .44 | 171.00 | .19 | .848 |
| BDI-2 × Induction focus × Time | -.03 | .03 | 171.00 | -1.14 | .257 | -.04 | .03 | 171.00 | -1.57 | .119 |

*Note*. BDI-2 = Beck Depression Inventory total score. ^*^ *p* < .05, ^**^ *p* < .01, ^***^ *p* < .001.

**Method S1. Recruitment and screening procedures**

Participants were recruited from a university-based undergraduate subject pool across five consecutive academic semesters (Spring 2016 through Spring 2018). Each semester, we screened an average of 922.80 students (*SD* = 79.94) yielding a total population of 4,614 individuals screened across the study period. Participants were classified into three mutually exclusive groups using prescreen GAD-Q-IV and BDI-II scores. The GAD group were required to meet GAD-Q-IV diagnostic criteria and scored ≤ 13 on the BDI-II; the Depression group scored ≥ 20 on the BDI-II and did not meet GAD-Q-IV criteria; and the low symptom comparison group (LS) did not meet GAD-Q-IV criteria and scored ≤ 13 on the BDI-II. Across the total subject pool (*N* = 4,614), 319 individuals (6.9%) met criteria for the GAD group, 343 individuals (7.4%) met criteria for the Depression group, and 2,899 individuals (62.8%) met criteria for the LS group. All eligible participants in the GAD and Depression groups were invited to participate in the study via email during each semester. For the LS group, recruitment was conducted using targeted email invitations to a fixed subset of eligible participants per semester in order to approximate the size of each clinical-symptom group and to maintain balanced group representation across experimental conditions. Of the eligible participants, a total of 356 individuals (7.7% of the overall subject pool) were enrolled in the present study. This included 118 participants from the GAD group (37.0% of eligible GAD participants), 113 participants from the Depression group (32.9% of eligible Depression participants), and 125 participants from the LS group (4.3% of eligible LS participants). Participation in the study was entirely voluntary, and students were free to decline participation or discontinue involvement at any time without penalty.

**Method S2. Definitions of worry and rumination**

Before we begin the experiment, I will explain the concepts of worry and rumination, as well as their differences.

***Definition of worry***

Worry is defined as a chain of uncontrollable thoughts, images, and doubts about things that might happen in the future. Worry is different from rumination because worry is concerned with potential negative events in the future, whereas, rumination focuses on negative feelings and experiences from the past.

Here are some examples of worry:

1. Worrying about not being attractive to others.
2. Worrying about making mistakes at work.
3. Worrying about not achieving one’s ambitions.
4. Worrying about financial problems that might restrict one’s life.

***Definition of rumination***

Rumination is defined as passively and repetitively thinking about the possible causes, implications, and consequences of stressful events and negative feelings as opposed to their solutions. Rumination is different from worry because rumination focuses on negative feelings and experiences from the past, whereas worry is concerned with potential negative events in the future.

Here are some examples of rumination:

1. Thinking over the loss of a close loved one
2. Rehashing in your mind recent things you’ve said or done
3. Spending a great deal of time thinking back over your embarrassing or disappointing moments
4. Keep going back to what happened long after an argument or disagreement is over with

**Method S3. Instructions for the personalized worry and rumination induction tasks**

***Personalized worry induction (practice version)***

Now, we will practice a one-minute self-administered induction. In this section, I want you to think about five topics that make you worry the most.

When you are ready, please think of five concerns that you currently worry about the most and are emotionally charged for you. Write them down on the paper provided by the experimenter. Rate how much you experienced each cognitive state as you thought about each concern. Once you have completed the rating, please return the paper to the experimenter and wait for further instructions.

*[Screen worrisome topics]*

Now you are ready to practice a one-minute self-administered induction. Please refer to your list of worrisome topics. When the experimenter instructs you to begin, close your eyes and think about your most worrisome topic in the way that you usually worry about it, but as intensely as you can. If you typically focus on only one topic at a time, try to do the same during this period. However, if your thoughts shift to another worrisome topic, feel free to let them continue. Please follow this process until you hear the beep and the experimenter instructs you to open your eyes.

If you are ready to begin, please click the mouse button.

***Personalized worry induction (experiment version)***

Good job! Now we are done practicing. We are ready to begin the experiment with a one-minute self-administered induction.

Please refer to your list of worrisome topics you made in the practice section. When the experimenter instructs you to begin, close your eyes and think about your most worrisome topic in the way that you usually worry about it, but as intensely as you can. If you typically focus on only one topic at a time, try to do the same during this period. However, if your thoughts shift to another worrisome topic, feel free to let them continue. Please follow this process until you hear the beep and the experimenter instructs you to open your eyes.

If you are ready to begin, please click the mouse button.

***Personalized rumination induction (practice version)***

Now, we will practice a one-minute self-administered induction. In this section, I want you to think about five topics that make you ruminate the most.

When you are ready, please think of five past experiences that you currently ruminate on the most and are emotionally charged for you. Write them down on the paper provided by the experimenter. Rate how much you experienced each cognitive state as you thought about each experience. Once you have completed the rating, please return the paper to the experimenter and wait for further instructions.

*[Screen ruminative topics]*

Now you are ready to practice a one-minute self-administered induction. Please refer to your list of ruminative topics. When the experimenter instructs you to begin, close your eyes and think about your most ruminative topic in the way that you usually ruminate on it, but as intensely as you can. If you typically focus on only one topic at a time, try to do the same during this period. However, if your thoughts shift to another ruminative topic, feel free to let them continue. Please follow this process until you hear the beep and the experimenter instructs you to open your eyes.

If you are ready to begin, please click the mouse button.

***Personalized rumination induction (experiment version)***

Good job! Now we are done practicing. We are ready to begin the experiment with a one-minute self-administered induction.

Please refer to your list of ruminative topics. When the experimenter instructs you to begin, close your eyes and think about your most ruminative topic in the way that you usually ruminate on it, but as intensely as you can. If you typically focus on only one topic at a time, try to do the same during this period. However, if your thoughts shift to another ruminative topic, feel free to let them continue. Please follow this process until you hear the beep and the experimenter instructs you to open your eyes.

If you are ready to begin, please click the mouse button.

***Worry/rumination topic entry form***

| Participant ID: ___________ |
| --- |
| Scenario 1 |
|  |
|  |
| Scenario 2 |
|  |
|  |
| Scenario 3 |
|  |
|  |
| Scenario 4 |
|  |
|  |
| Scenario 5 |
|  |

***Screening measures for the personalized worry/rumination induction methods***

Below are cognitive states that you can experience from each of the scenarios. Please rate by circling the number that best describes your cognitive experience from each of the scenarios.

**Scenario 1**

How worrisome is this scenario for you?

0 ------------ 1 ------------ 2 ------------ 3 ------------ 4 ------------ 5 ------------ 6 ------------ 7 ------------ 8

Not at all Somewhat Extremely

How ruminative is this scenario for you?

0 ------------ 1 ------------ 2 ------------ 3 ------------ 4 ------------ 5 ------------ 6 ------------ 7 ------------ 8

Not at all Somewhat Extremely

How relaxing is this scenario for you?

0 ------------ 1 ------------ 2 ------------ 3 ------------ 4 ------------ 5 ------------ 6 ------------ 7 ------------ 8

Not at all Somewhat Extremely

When you were thinking about this scenario, where were you inclined to focus your attention?

0 ------------ 1 ------------ 2 ------------ 3 ------------ 4 ------------ 5 ------------ 6 ------------ 7 ------------ 8

Past Present Future

**Scenario 2**

How worrisome is this scenario for you?

0 ------------ 1 ------------ 2 ------------ 3 ------------ 4 ------------ 5 ------------ 6 ------------ 7 ------------ 8

Not at all Somewhat Extremely

How ruminative is this scenario for you?

0 ------------ 1 ------------ 2 ------------ 3 ------------ 4 ------------ 5 ------------ 6 ------------ 7 ------------ 8

Not at all Somewhat Extremely

How relaxing is this scenario for you?

0 ------------ 1 ------------ 2 ------------ 3 ------------ 4 ------------ 5 ------------ 6 ------------ 7 ------------ 8

Not at all Somewhat Extremely

When you were thinking about this scenario, where were you inclined to focus your attention?

0 ------------ 1 ------------ 2 ------------ 3 ------------ 4 ------------ 5 ------------ 6 ------------ 7 ------------ 8

Past Present Future

**Scenario 3**

How worrisome is this scenario for you?

0 ------------ 1 ------------ 2 ------------ 3 ------------ 4 ------------ 5 ------------ 6 ------------ 7 ------------ 8

Not at all Somewhat Extremely

How ruminative is this scenario for you?

0 ------------ 1 ------------ 2 ------------ 3 ------------ 4 ------------ 5 ------------ 6 ------------ 7 ------------ 8

Not at all Somewhat Extremely

How relaxing is this scenario for you?

0 ------------ 1 ------------ 2 ------------ 3 ------------ 4 ------------ 5 ------------ 6 ------------ 7 ------------ 8

Not at all Somewhat Extremely

When you were thinking about this scenario, where were you inclined to focus your attention?

0 ------------ 1 ------------ 2 ------------ 3 ------------ 4 ------------ 5 ------------ 6 ------------ 7 ------------ 8

Past Present Future

**Scenario 4**

How worrisome is this scenario for you?

0 ------------ 1 ------------ 2 ------------ 3 ------------ 4 ------------ 5 ------------ 6 ------------ 7 ------------ 8

Not at all Somewhat Extremely

How ruminative is this scenario for you?

0 ------------ 1 ------------ 2 ------------ 3 ------------ 4 ------------ 5 ------------ 6 ------------ 7 ------------ 8

Not at all Somewhat Extremely

How relaxing is this scenario for you?

0 ------------ 1 ------------ 2 ------------ 3 ------------ 4 ------------ 5 ------------ 6 ------------ 7 ------------ 8

Not at all Somewhat Extremely

When you were thinking about this scenario, where were you inclined to focus your attention?

0 ------------ 1 ------------ 2 ------------ 3 ------------ 4 ------------ 5 ------------ 6 ------------ 7 ------------ 8

Past Present Future

**Scenario 5**

How worrisome is this scenario for you?

0 ------------ 1 ------------ 2 ------------ 3 ------------ 4 ------------ 5 ------------ 6 ------------ 7 ------------ 8

Not at all Somewhat Extremely

How ruminative is this scenario for you?

0 ------------ 1 ------------ 2 ------------ 3 ------------ 4 ------------ 5 ------------ 6 ------------ 7 ------------ 8

Not at all Somewhat Extremely

How relaxing is this scenario for you?

0 ------------ 1 ------------ 2 ------------ 3 ------------ 4 ------------ 5 ------------ 6 ------------ 7 ------------ 8

Not at all Somewhat Extremely

When you were thinking about this scenario, where were you inclined to focus your attention?

0 ------------ 1 ------------ 2 ------------ 3 ------------ 4 ------------ 5 ------------ 6 ------------ 7 ------------ 8

Past Present Future

***Selection criteria for worry/rumination topics***

1. Target cognitive state should be higher than or equal to 5.
2. Target cognitive state should be at least 3 points higher than non-target state.
   e.g., To be eligible for a worry induction: worry 7, rumination 4, relaxation 4
3. The temporal orientation of the worry scenario should be higher than or equal to 5. Conversely, the temporal orientation of the rumination scenario should be lower than or equal to 3.
4. If no scenario meets all inclusion criteria, the participant is not eligible to proceed with the personalized induction. If multiple scenarios meet all inclusion criteria and receive identical highest scores, all qualifying scenarios should be retained. During the induction phase, participants should be instructed to select one of these equally qualifying scenarios as the focus of the induction.

Erase unselected scenarios. There should be at least one scenario remaining on the list. During the induction phase, ask participants to engage in their most emotionally charged scenarios.

**Method S4. Instructions for the scripted worry and rumination induction tasks**

***Scripted Worry Induction***^[[1]](#footnote-1)^

For the next few minutes, try your best to focus your attention on each of the ideas on the following pages. Read each item slowly and silently to yourself. As you read the items, use your imagination and concentration to focus your mind on each of the ideas. Spend a few moments visualizing and concentrating on each item. Once you have fully engaged in each item, click the mouse button to proceed to the next item. Please continue until you hear the beep.

Think about the following…

1. My money will run out.
2. I won’t be able to be assertive or express my opinions.
3. My future job prospects will not be good.
4. My family will be angry with me or disapprove of something that I do.
5. I will never achieve my ambitions.
6. I will not keep up with my work.
7. Financial problems will restrict holidays and travel.
8. I will not be able to concentrate.
9. I will not have enough money to afford things.
10. I will develop insecurities.
11. I will not be able to afford to pay bills in the future.
12. My living conditions will be inadequate.
13. My life will have no purpose.
14. I will not be able to work hard enough.
15. Others will not approve of me.
16. I will find it difficult to maintain a stable relationship.
17. I will not be able to finish my work.
18. I will lack confidence.
19. I will not be attractive to others.
20. I might make myself look stupid in front of others.
21. I will lose my close friends.
22. I will not be able to achieve my goals.
23. I will not be loved.
24. I will be late for an appointment.
25. I will make mistakes at work.

***Scripted Rumination Induction***^[[2]](#footnote-2)^

For the next few minutes, do your best to focus your attention on each of the ideas on the following pages. Read each item slowly and silently to yourself. As you read the items, use your imagination and concentration to focus your mind on each of the ideas. Spend a few moments visualizing and concentrating on each item. Once you have fully engaged with an item, click the mouse button to proceed to the next one. Please continue until you hear the beep.

Think about…

1. the physical sensations you feel in your body
2. your character and who you strive to be
3. the degree of clarity in your thinking right now
4. why you react the way you do
5. the way you feel inside
6. the possible consequences of your current mental state
7. how similar/different you are relative to other people
8. what it would be like if your present feelings lasted
9. why things turn out the way they do
10. trying to understand your feelings
11. how awake/tired you feel now
12. the amount of tension in your muscles
13. whether you are fulfilled
14. your physical appearance
15. whether you feel stressed right now
16. the long-term goals you have set
17. the amount of certainty you feel
18. your present feelings of fatigue/energy
19. possible explanations for your physical sensations
20. how hopeful/hopeless you are feeling
21. the level of motivation you feel right now
22. the degree of helplessness you feel
23. the degree of calmness/restlessness you feel
24. the possible consequences of the way you feel
25. what your feelings might mean
26. how sad/happy you are feeling
27. the expectations your family has for you
28. why your body feels this way
29. why you get this way sometimes
30. how passive/active you feel
31. what people notice about your personality
32. the kind of student you are and wish you were
33. how weak/strong your body feels now
34. the degree of relaxation/agitation you feel
35. the kind of person you think you should be
36. the degree of control you feel right now
37. what would happen if your current physical state lasted
38. sitting down and analyzing your personality
39. why you turned out this way
40. the things that are most important in your life
41. how quick/slow your thinking is right now
42. the degree of decisiveness you feel
43. trying to understand who you are
44. how you feel about your friendships
45. whether you have accomplished a lot so far

1. These items are adapted from the Worry Domains Questionnaire developed by Tallis, Davey, & Bond (1994), who retain the rights to the original content. [↑](#footnote-ref-1)
2. This script is adapted from the Ruminative Response Task developed by Nolen-Hoeksema & Morrow (1993), who retain rights to the original focus. [↑](#footnote-ref-2)
